# Ninety‐Day Readmission and Morbidity Following Liver Transplantation for MASLD

**DOI:** 10.1111/ctr.70461

**Published:** 2026-02-04

**Authors:** Zachary Leslie, Thomas Leventhal, Sean Nguyen, David Leishman, Cheyenna Espinoza, Eric Wise, Sayeed Ikramuddin, Raja Kandaswamy, Abraham J. Matar

**Affiliations:** ^1^ Carleton College Northfield Minnesota USA; ^2^ Department of Medicine, Division of Gastroenterology, Hepatology, and Nutrition University of Minnesota Minneapolis Minnesota USA; ^3^ Department of Surgery, Division of Gastrointestinal/Bariatric Surgery University of Minnesota Minneapolis Minnesota USA; ^4^ Department of Surgery, Division of Transplantation University of Minnesota Minneapolis Minnesota USA

**Keywords:** liver transplantation, metabolic dysfunction‐associated steatotic liver disease, morbidity, readmission

## Abstract

**Introduction:**

The incidence of short‐term (90‐day) hospital readmission and morbidity following liver transplantation (LT) for metabolic dysfunction‐associated steatotic liver disease (MASLD) is poorly characterized, and no study to date has distinguished these short‐term outcomes between MASLD and non‐MASLD LT recipients. The primary objective of this study was to leverage the National Readmissions Database (NRD) to distinguish 90‐day readmission rates and morbidity among those undergoing LT for MASLD and non‐MASLD etiologies.

**Methods:**

Recipients undergoing LT were identified in the NRD between January 1, 2016, and December 31, 2022. Morbidity was defined as an aggregate of common surgical complications. Univariate and age‐sex adjusted quasi‐Poisson regressions were used to identify trends and differences in characteristics for patients stratified by indication for LT. Multivariable logistic regression models were used to identify factors associated with 90‐day readmissions and morbidity.

**Results:**

A weighted total of 58 148 LT procedures were identified, of which 11 235 (19.3%) had MASLD etiology. LT for MASLD increased over the study period, while LT for hepatitis C and liver cancer decreased. Relative to non‐MASLD LT recipients, MASLD LT recipients had increased comorbid risk profiles, including higher rates of class 3 obesity (body mass index [BMI] ≥ 40) and associated liver cancer. MASLD LT recipients had lower rates of 90‐day readmission, morbidity, and cardiovascular complications (all *p* < 0.05). Among patients with class 3 obesity and liver cancer, MASLD etiology was associated with improved or non‐inferior 90‐day outcomes relative to non‐MASLD LT recipients. Finally, among MASLD LT recipients, the presence of chronic kidney disease, prior bariatric surgery, dialysis, female sex, and chronic obstructive pulmonary disease were independent predictors of 90‐day morbidity and readmission.

**Conclusions:**

Despite an increased comorbid risk profile, MASLD LT recipients had superior 90‐day readmission rates and morbidity outcomes relative to non‐MASLD LT recipients. MASLD etiology appears to normalize 90‐day outcomes among patients with obesity and liver cancer.

**Abbreviations**
CKD: chronic kidney disease
COPD: chronic obstructive pulmonary disease
DVT: deep vein thrombosis
ESLD: end‐stage liver disease
HCUP: Healthcare Cost and Utilization Project
ICD: International Classification of Diseases
IRR: incidence risk ratios
LT: liver transplantation
MASLD: metabolic dysfunction‐associated steatotic liver disease
MELD: model for end‐stage liver disease
MI: myocardial infarction
NAFLD: non‐alcoholic fatty liver disease
NRD: national readmissions database
OR: odds ratio
PE: pulmonary embolism

## 1 Introduction

Metabolic dysfunction‐associated steatotic liver disease (MASLD) represents a rapidly increasing indication for liver transplantation (LT) and is currently the second most common indication for LT behind alcohol associated liver disease [1, 2, 3, 4, 5]. MASLD is closely tied to obesity and the associated metabolic syndrome [6, 7]. However, the association between obesity, MASLD, and post‐LT outcomes is not entirely clear, in part due to the obesity paradox, which refers to the observation that increasing obesity is associated with improved survival outcomes after LT. This paradox creates difficulty when gauging the relative risk of obesity and associated metabolic comorbidities in LT [8, 9, 10].

Patients with MASLD‐associated end‐stage liver disease (ESLD) have different risk profiles than those with non‐MASLD ESLD, namely higher rates of metabolic syndrome‐associated comorbidities [11, 12]. Currently, the incidence of short‐term (90‐day) hospital readmission and morbidity following LT for MASLD is poorly characterized, and no study to date has distinguished these short‐term outcomes between MASLD and non‐MASLD LT recipients. As obesity rates continue to increase, understanding the impact of MASLD‐associated comorbidities on postoperative outcomes is critical to identifying opportunities to improve outcomes. The Nationwide Readmissions Database (NRD) is a representative sample of the entire US surgical population and contains information on patient readmissions through unique admission‐linking identifiers [13, 14]. The purpose of this study was to leverage the NRD to distinguish 90‐day readmission rates and morbidity among those undergoing LT for MASLD and non‐MASLD etiologies.

## 2 Methods

### 2.1 Data Source

The Healthcare Cost and Utilization Project (HCUP) NRD database is a representative sampling of surgeries and readmission events in the US. The NRD samples from 30 state databases, and patients are linked using unique identifier numbers to identify connected readmissions. The core and hospital files of the NRD were merged and combined between January 1, 2016, and December 31, 2022 [15]. The 10th revision of the International Statistical Classification of Diseases and Related Health Problems (ICD‐10) was used to identify LT procedures (Table S1) [16]. Common comorbidities for patients with liver disease were identified using appropriate ICD‐10‐CM codes in the NRD. Inclusion and exclusion criteria are outlined in a CONSORT flow diagram (Figure S1). Importantly, in this study, the diagnosis of “nonalcoholic fatty liver disease” (NAFLD) was used in the search criteria rather than MASLD. MASLD represents an updated nomenclature that was widely adopted in 2023 in an effort to eliminate the potentially stigmatizing terms “nonalcoholic” and “fatty ” [17].

This study was deemed exempt from the International Review Board (IRB) review because of the de‐identified, retrospective nature of the dataset used. This study adheres to the Strengthening the Reporting of Observational Studies in Epidemiology (STROBE) and the HCUP Agency for Healthcare Research and Quality (AHRQ) reporting guidelines [18].

### 2.2 Preoperative and Perioperative Factors

Demographic variables, including age, sex, insurance‐payer status, and year of operation, were gathered from header labels in the NRD. The estimated median household income of residents in the patient's zip code was collected from the ZIPINC_QRTL variable, which categorizes the counties in which patients live from poorest to wealthiest populations (scored 1–4). The county size of residence was gathered from PL_NCHS, which designates urban and rural regions with codes ranging from 1, representing a larger metropolitan county, to 6, representing a non‐metropolitan or non‐micropolitan county. Hospital characteristics, including bed size and hospital volume, were included and calculated, respectively, from the hospital file of the NRD. Hospital volume was determined by a count of unique hospital identifiers for an index admission in each hospital; high‐volume centers were defined as those which had 90 or more cases from accepted hospital volume classifications for patients undergoing LT [19]. The Charlson comorbidity index was included as an independent risk factor, as it is a validated scoring method for postoperative patient outcomes after surgery [20].

### 2.3 Postoperative Factors

Ninety‐day postoperative morbidity and 90‐day readmission after hospital discharge were the primary outcome variables of choice in this study. These events were identified with appropriate ICD‐10 codes and from the NRD_VISITLINK and NRD_DAYSTOEVENT data fields. Ninety‐day readmission from the time of discharge was calculated from NRD_VISITLINK, NRD_DAYSTOEVENT, and LOS. Ninety‐day morbidity was defined as the aggregate of acute renal failure, sepsis, blood transfusion, urinary tract infection, pulmonary embolism (PE), deep vein thrombosis (DVT), cardiac arrest, myocardial infarction (MI), infection, ventilator dependency, and pneumonia occurring either during the index or any subsequent hospitalization within 90 days of operation. A subgroup analysis looking at cardiovascular complications was defined as the aggregate of PE, DVT, cardiac arrest, and MI.

### 2.4 Statistical Analysis

Within the NRD, factors in the groups with and without MASLD were compared univariately with binomial logistic regressions to determine differences between the cohorts while accounting for sample statistical weights. Body mass index (BMI) was imputed using a k‐nearest neighbors algorithm with five nearest neighbors because it was 15% missing for patients with class III or higher obesity and 62% missing for patients without class III or higher obesity. The covariates age, female sex, diabetes, and class III or higher obesity status were used to determine the nearest neighbors. Continuous variables in the characteristic table were represented as mean ±standard deviation. Quasi‐Poisson age‐sex adjusted regression models determined trends in major diagnosis for LT as incidence risk ratios (IRRs), which included liver cancer, alcoholic cirrhosis of the liver, MASLD, hepatitis C, and other diagnosis for LT. Rows were removed if they had a missing value for any of the variables used in this study [21]. Continuous variables were standardized by subtracting the mean of the column from each value and dividing the result by the standard deviation of the column for regression analysis. Trends in readmission and morbidity rates were analyzed using single‐subject correlation with 95% confidence intervals. Independent risk factors were identified, and a multivariable binomial logistic regression model was developed. Alcoholic cirrhosis of the liver, large hospital bed size, and Medicare payer status were used as reference variables for LT etiology, hospital bed size, and insurance payer categorizations, respectively, to determine the adjusted risk of MASLD indication. All demographic, hospital/operative factors, and comorbid factors were used to adjust risk, which included all of those in the characteristic tables except the reference variables. For the purposes of this study, *p* < 0.05 was deemed statistically significant when comparing the two cohorts and to determine significance for each individual risk factor in the regression model. Python (v. 3.12) and the pandas, polars, and scikit‐learn libraries were used for all statistical analyses.

## 3 Results

### 3.1 MASLD Recipient Characteristics

**FIGURE 1:**
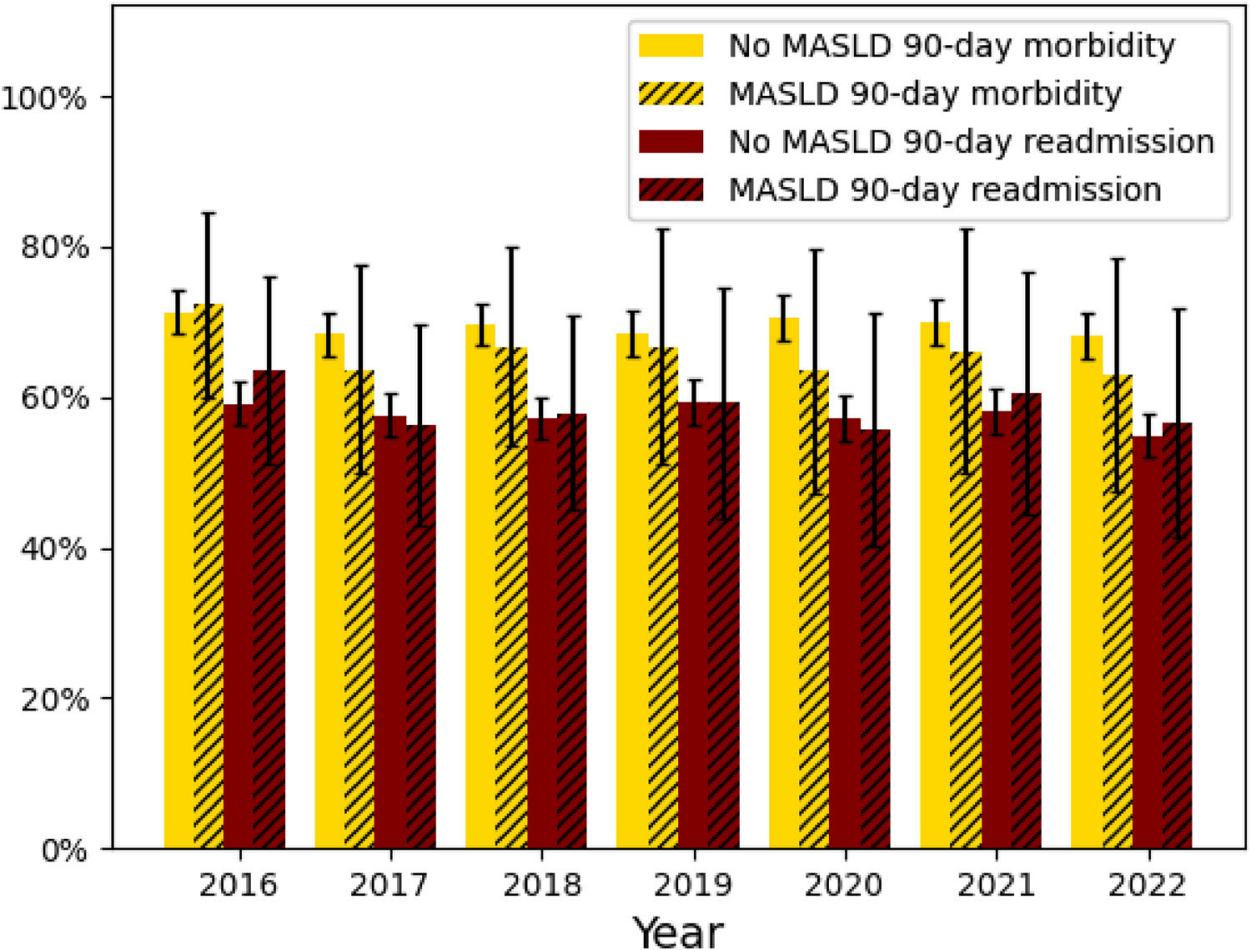
Rates of 90‐day readmission and morbidity stratified by MASLD recipient indication within the NRD between 2016 and 2022. Correlation adjusted error bars used to denote uncertainty from sampling methodology.

A weighted total of 58 148 LT procedures were identified, of which 11 235 (19.3%) had a primary diagnosis of MASLD. The overall rate of 90‐day readmission and morbidity in MASLD LT recipients was 58.1% and 67.3%, respectively, compared to 57.5% and 70.5% (*p* < 0.05) for non‐MASLD LT recipients (Figure 1). Adverse cardiovascular events occurred less frequently in MASLD LT recipients (12.7% vs. 13.8%, *p* < 0.05). Over the study period, the incidence of LT for MASLD increased by 57%, which represented the most significant increase (IRR: 1.2, *p* < 0.05, Table 1), followed by LT for alcohol associated liver disease (IRR: 1.1, *p* < 0.05). LT for hepatitis C (IRR: 0.7, *p* < 0.05), liver cancer (IRR: 0.9, *p* < 0.05), and other diagnoses (IRR: 0.9, *p* < 0.05) decreased over the study period.

**TABLE 1:** Trends in etiologies among recipients undergoing LT within the NRD between 2016 and 2022.

Year	Alcohol‐associated liver disease	HCV	Liver cancer	MASLD	Other
2016	2253	2027	1820	1122	2655
2017	2476	1968	2012	1387	2463
2018	2120	1389	1656	1278	2263
2019	3121	1325	1834	1811	2710
2020	2294	1130	1727	1922	2567
2021	3858	920	1571	1945	2638
2022	3642	809	1471	1770	2313
Incidence risk ratio	1.11^*^	0.73^*^	0.94^*^	1.23^*^	0.94^*^
Adjusted risk 90‐day morbidity (OR [95% CI])	Reference	0.69 [0.65, 0.73] ^*^	0.50 [0.47, 0.54] ^*^	0.84 [0.79, 0.89] ^*^	0.94 [0.89, 0.99] ^*^
Adjusted risk 90‐day readmission (OR [95% CI])	Reference	0.81 [0.77, 0.86] ^*^	0.50 [0.47, 0.53] ^*^	0.93 [0.88, 0.99] ^*^	0.88 [0.83, 0.93] ^*^

* Denotes significant OR or IRR.

Abbreviations: HCV, hepatitis C; MASLD, metabolic dysfunction‐associated steatotic liver disease.

Relative to non‐MASLD LT recipients, MASLD LT recipients were older, more often female, had a higher incidence of class 3 obesity (BMI ≥ 40), were Medicare payers, from more rural areas and had increased comorbidity risk profiles as indicated by higher Charlson comorbidity scores (Table 2). MASLD LT recipients more often travelled to different states for their operations, more frequently underwent operations in higher volume centers, and had decreased index hospital charges. Additionally, the recipients less frequently had metabolic dysfunction‐associated alcohol‐related liver disease.

**TABLE 2:** Characteristics and outcomes for MASLD versus non‐MASLD etiology within the NRD between 2016 and 2022.

Variable	Non‐MASLD etiology (*n* = 46 913)	MASLD etiology (*n* = 11 235)	*p* value
**Demographics**			
Age (years)	49.59 (±17.71)	60.07 (±8.43)	< 0.05
Female sex	16 394 (34.94%)	4941 (43.98%)	< 0.05
BMI (kg/m^2^)	29.96 (±5.42)	33.17 (±5.98)	< 0.05
**Hospital/operative factors**			
High volume (>90 cases annually)	15 173 (32.34%)	4303 (38.30%)	< 0.05
Elective	6199 (13.21%)	1791 (15.94%)	< 0.05
**Comorbidities**			
Charlson comorbidity index	2.32 (±1.75)	3.16 (±1.91)	< 0.05
Chronic heart failure	2392 (5.10%)	837 (7.45%)	< 0.05
Coronary heart disease	3422 (7.29%)	1626 (14.47%)	< 0.05
COPD	1733 (3.69%)	420 (3.74%)	< 0.05
Presence coronary graft	992 (2.11%)	575 (5.12%)	< 0.05
CKD	9108 (19.41%)	2892 (25.74%)	< 0.05
Hypertension	14 580 (31.08%)	4265 (37.96%)	< 0.05
Hyperlipidemia	6297 (13.42%)	3609 (32.12%)	< 0.05
Class III or higher obesity	1794 (3.82%)	1288 (11.46%)	< 0.05
Metabolic dysfunction‐associated alcohol‐related liver disease	848 (1.81%)	136 (1.21%)	< 0.05
Smoker	3339 (7.12%)	296 (2.63%)	< 0.05
Type 2 diabetes	10 716 (22.84%)	6586 (58.62%)	< 0.05
Prior bariatric surgery	654 (1.39%)	284 (2.53%)	< 0.05
Dialysis	1660 (3.54%)	379 (3.37%)	0.99
**Outcomes**			
Readmission	26 989 (57.53%)	6529 (58.11%)	0.17
Morbidity	33 094 (70.54%)	7566 (67.34%)	< 0.05
Mortality in‐hospital index	765 (1.63%)	141 (1.26%)	< 0.05
Acute renal failure	1102 (2.35%)	302 (2.69%)	0.14
Sepsis	13 232 (28.20%)	2524 (22.47%)	< 0.05
Blood transfusion	19 378 (41.31%)	4344 (38.66%)	< 0.05
UTI	7336 (15.64%)	2175 (19.36%)	< 0.05
PE	4564 (9.73%)	960 (8.54%)	< 0.05
DVT	815 (1.74%)	135 (1.2%)	< 0.05
Cardiovascular event	6482 (13.82%)	1423 (12.67%)	< 0.05
Cardiac arrest	784 (1.67%)	153 (1.36%)	< 0.05
MI	1173 (2.50%)	349 (3.11%)	< 0.05
Infection	15 165 (32.33%)	3452 (30.73%)	< 0.05
Ventilator dependency	9915 (21.13%)	2116 (18.83%)	< 0.05
Pneumonia	6755 (14.40%)	1404 (12.50%)	< 0.05
Home discharge after transplantation	29 065 (62.0%)	6336 (56.4%)	< 0.05

Abbreviations: BMI, body mass index; CI, confidence interval; CKD, chronic kidney disease; COPD, chronic obstructive pulmonary disease; DVT, deep vein thrombosis; GERD, gastroesophageal reflux disease; IRR, incidence risk ratio; MASLD, metabolic dysfunction‐associated steatotic liver disease; MI, myocardial infarction; NSAID, non‐steroidal anti‐inflammatory drug; OR, odds ratio; PE, pulmonary embolism; UTI, urinary tract infection; all complications are within 90‐days from inpatient liver transplant surgery.

### 3.2 MASLD Recipient Outcomes

MASLD LT recipients experienced shorter lengths of stay (12.9 vs. 16.7 days, *p* < 0.05), decreased in‐hospital mortality (1.2% vs. 1.6%, *p* < 0.05), decreased 90‐day postoperative morbidity (67.3% vs. 70.5%, *p* < 0.05), decreased rates of cardiovascular‐related complications (12.67% vs. 13.82%, *p* < 0.05), and similar 90‐day readmission rates (58.1% vs. 57.5%, *p* = 0.17 (Table 2) compared to non‐MASLD LT recipients. After adjusting for independent health factors, MASLD was associated with a significantly decreased risk of both 90‐day readmission (OR: 0.93 [0.87,0.98], *p* < 0.05) and 90‐day morbidity (OR: 0.83 [0.79, 0.89], *p* < 0.05).

Among MASLD LT recipients, adjusted multivariate regression analysis identified chronic kidney disease (CKD), prior bariatric surgery, dialysis, female sex, and chronic obstructive pulmonary disease (COPD) as predictive of 90‐day morbidity and readmission. Conversely, increasing Charlson comorbidity index scores, LT at a high‐volume center, and long‐term non‐steroidal anti‐inflammatory drug usage were associated with a decreased risk in both outcomes.

### 3.3 MASLD Recipients Stratified by Obesity

Among MASLD LT recipients, 1319/11 234 (11.7%) of patients had class 3 obesity (BMI ≥ 40) (Table 3, Figure S2). Relative to MASLD LT recipients with BMI < 40, those with BMI ≥ 40 had a higher incidence of metabolic‐syndrome related comorbidities, including chronic heart failure, coronary artery disease, chronic kidney disease, hyperlipidemia, and need for dialysis. Those with a BMI ≥ 40 also had a higher incidence of prior bariatric surgery. Despite an increased rate of comorbidities, when comparing 90‐day outcomes, MASLD recipients with BMI ≥ 40 had similar rates of 90‐day readmission (59.6% vs. 58.2%, *p* = 0.3), cardiovascular events (12.4% vs. 12.7%, *p* = 0.78), and mortality (1.4% vs. 1.4%, *p* = 0.86), and had a decreased incidence of 90‐day morbidity (62.3% vs. 66.1%, *p* < 0.05) (Table 3).

**TABLE 3:** Characteristics and outcomes for recipients undergoing LT for MASLD stratified by obesity status within the NRD between 2016 and 2022.

Variable	BMI < 40 (*n* = 9915)	BMI ≥ 40 (*n* = 1319)	*p* value
**Demographics**			
Age (years)	60.53 (±8.26)	56.67 (±8.9)	< 0.05
Female sex	4327 (43.64%)	571 (43.29%)	0.82
BMI (kg/m^2^)	31.99 (±4.91)	42.05 (±5.79)	< 0.05
**Hospital/operative factors**			
High volume (>90 cases annually)	3927 (39.60%)	519 (39.34%)	0.88
Elective	1567 (15.80%)	200 (15.16%)	0.57
**Comorbidities**			
Charlson comorbidity index	3.15 (±1.91)	3.19 (±1.91)	0.60
Chronic heart failure	724 (7.30%)	125 (9.48%)	< 0.05
Coronary heart disease	1511 (15.24%)	151 (11.45%)	<0.05
COPD	403 (4.06%)	57 (4.32%)	0.63
Presence coronary graft	573 (5.78%)	48 (3.64%)	< 0.05
CKD	2581 (26.03%)	381 (28.88%)	< 0.05
Hypertension	3723 (37.55%)	493 (37.37%)	0.92
Hyperlipidemia	3205 (32.32%)	384 (29.11%)	< 0.05
Smoker	320 (3.23%)	22 (1.67%)	< 0.05
Type 2 diabetes	5910 (59.60%)	742 (56.25%)	< 0.05
Prior bariatric surgery	213 (2.15%)	55 (4.17%)	< 0.05
Dialysis	317 (3.20%)	61 (4.62%)	< 0.05
**Outcomes**			
Readmission	5766 (58.15%)	786 (59.59%)	0.30
Morbidity	6551 (66.07%)	822 (62.32%)	< 0.05
Mortality in‐hospital index	134 (1.35%)	18 (1.36%)	0.86
Acute renal failure	242 (2.44%)	30 (2.27%)	0.80
Sepsis	2146 (21.64%)	279 (21.15%)	0.71
Infection	3036 (30.62%)	398 (30.17%)	0.75
Blood transfusion	3560 (35.90%)	447 (33.89%)	0.16
UTI	1944 (19.60%)	247 (18.72%)	0.48
PE	815 (8.22%)	109 (8.26%)	0.89
DVT	131 (1.32%)	6 (0.45%)	< 0.05
Cardiovascular event	1259 (12.7%)	163 (12.36%)	0.78
Cardiac arrest	141 (1.42%)	21 (1.59%)	0.61
MI	319 (3.22%)	31 (2.35%)	0.10
Ventilator dependency	1929 (19.45%)	203 (15.39%)	< 0.05
Pneumonia	1247 (12.58%)	132 (10.01%)	< 0.05
LOS	12.90 (±17.68)	12.45 (±17.21)	0.54
Home discharge after transplantation	5600 (56.47%)	735 (55.72%)	0.63

Abbreviations: BMI, body mass index; CI, confidence interval; CKD, chronic kidney disease; COPD, chronic obstructive pulmonary disease; DVT, deep vein thrombosis; GERD, gastroesophageal reflux disease; IRR, incidence risk ratio; MASLD, metabolic dysfunction‐associated steatotic liver disease; MI, myocardial infarction; NSAID, non‐steroidal anti‐inflammatory drug; OR, odds ratio; PE, pulmonary embolism; UTI, urinary tract infection; all complications are within 90‐days from inpatient liver transplant surgery.

We then compared outcomes between MASLD versus non‐MASLD LT recipients with BMI ≥ 40 (Table 4). MASLD LT recipients with BMI ≥ 40 were older, more likely to be female, and had higher Charlson comorbidity index scores. Relative to non‐MASLD LT recipients with BMI ≥ 40, MASLD LT recipients with BMI ≥ 40 had higher rates of coronary artery disease, chronic kidney disease, hyperlipidemia, and type 2 diabetes mellitus. Despite a higher comorbid risk profile, MASLD LT recipients with BMI ≥ 40 had superior outcomes, including decreased length of stay (12.5 days vs. 17.7 days, *p* < 0.05), and a decreased risk of 90‐day morbidity (62.3% vs. 73.8%, *p* < 0.05) and 90‐day cardiovascular events (12.4% vs. 15.8%, *p* < 0.05). Ninety‐day readmissions and overall mortality were not different.

**TABLE 4:** Characteristics and outcomes among LT recipients with a diagnosis of class 3 obesity (BMI > 40) stratified by MASLD vs. non‐MASLD within the NRD between 2016 and 2022.

Variable	Non‐MASLD (*n* = 1779)	MASLD (*n* = 1319)	*p* value
**Demographics**			
Age (years)	52.31 (±11.47)	56.67 (±8.90)	< 0.05
Female sex	676 (37.99%)	571 (43.29%)	< 0.05
BMI (kg/m^2^)	42.26 (±6.08)	42.05 (±5.79)	0.26
**Hospital/operative factors**			
High volume (>90 cases annually)	496 (27.88%)	519 (39.34%)	< 0.05
Elective	187 (10.51%)	200 (15.16%)	< 0.05
**Comorbidities**			
Charlson comorbidity index	2.75 (±1.87)	3.19 (±1.91)	< 0.05
Chronic heart failure	149 (8.37%)	125 (9.48%)	0.27
Coronary artery disease	152 (8.54%)	151 (11.45%)	< 0.05
COPD	86 (4.83%)	57 (4.32%)	0.50
Presence coronary graft	35 (1.97%)	48 (3.64%)	< 0.05
CKD	402 (22.59%)	381 (28.88%)	< 0.05
Hypertension	685 (38.50%)	493 (37.37%)	0.53
Hyperlipidemia	340 (19.11%)	384 (29.11%)	< 0.05
Smoker	151 (8.49%)	22 (1.67%)	< 0.05
Type 2 diabetes	606 (34.06%)	742 (56.25%)	< 0.05
Prior bariatric surgery	86 (4.83%)	55 (4.17%)	0.39
Dialysis	55 (3.09%)	61 (4.62%)	< 0.05
**Outcomes**			
Readmission	1084 (60.92%)	786 (59.59%)	0.47
Morbidity	1313 (73.79%)	822 (62.32%)	< 0.05
Mortality	34 (1.91%)	18 (1.36%)	0.25
Acute renal failure	60 (3.37%)	30 (2.27%)	0.08
Sepsis	560 (31.47%)	279 (21.15%)	< 0.05
Infection	647 (36.36%)	398 (30.17%)	< 0.05
Blood transfusion	713 (40.07%)	447 (33.89%)	< 0.05
UTI	358 (20.12%)	247 (18.72%)	0.34
PE	194 (10.90%)	109 (8.26%)	< 0.05
DVT	39 (2.19%)	6 (0.45%)	< 0.05
Cardiovascular event	281 (15.79%)	163 (12.36%)	< 0.05
Cardiac arrest	39 (2.19%)	21 (1.59%)	0.24
MI	42 (2.36%)	31 (2.35%)	0.95
Ventilator dependency	417 (23.44%)	203 (15.39%)	< 0.05
Pneumonia	317 (17.82%)	132 (10.01%)	< 0.05
LOS	17.69 (±23.50)	12.45 (±17.21)	< 0.05
Home discharge after transplantation	1011 (56.82%)	735 (55.72%)	0.56

Abbreviations: BMI, body mass index; CI, confidence interval; CKD, chronic kidney disease; COPD, chronic obstructive pulmonary disease; DVT, deep vein thrombosis; GERD, gastroesophageal reflux disease; IRR, incidence risk ratio; MASLD, metabolic dysfunction‐associated steatotic liver disease; MI, myocardial infarction; NSAID, non‐steroidal anti‐inflammatory drug; OR, odds ratio; PE, pulmonary embolism; UTI, urinary tract infection; all complications are within 90‐days from inpatient liver transplant surgery.

### 3.4 MASLD Recipients Stratified by Presence of Malignancy

Among LT recipients with MASLD, 2376/11 234 (21.2%) had an associated liver cancer diagnosis (Table S2). MASLD LT recipients with liver cancer were older and more frequently male. MASLD LT recipients with liver cancer had higher Charlson comorbidity scores, increased rates of coronary artery disease, hypertension, hyperlipidemia, and type 2 diabetes mellitus. When comparing 90‐day outcomes, MASLD LT recipients with liver cancer had decreased rates of length of stay, 90‐day readmission, 90‐day cardiovascular events, and 90‐day morbidity (all *p* < 0.05).

Finally, we compared outcomes between MASLD versus non‐MASLD LT recipients with a diagnosis of liver cancer (Table S3). MASLD LT recipients with liver cancer were older and more frequently female. Relative to non‐MASLD LT recipients with liver cancer, MASLD LT recipients with liver cancer had higher Charlson comorbidity scores, increased rates of coronary artery disease, hypertension, hyperlipidemia, and type 2 diabetes mellitus. When comparing 90‐day outcomes, MASLD LT recipients with liver cancer experienced a decreased length of stay (*p* < 0.05) and a strong trend towards reduced 90‐day morbidity and mortality (*p* = 0.05). Rates of 90‐day readmission and 90‐day cardiovascular events were similar.

## 4 Discussion

We utilized the NRD to study 90‐day outcomes among LT recipients and distinguished outcomes among MASLD and non‐MASLD etiologies. Relative to non‐MASLD LT recipients, those with MASLD represented a more at‐risk population, who were older, less socioeconomically affluent, and had greater comorbid risk profiles. Despite this, 90‐day outcomes were generally superior to non‐MASLD LT recipients. These findings held true in subset analyses of patients with class 3 obesity and a concomitant diagnosis of liver cancer. These findings highlight the association between a diagnosis of MASLD and improved short‐term outcomes following LT.

Understanding the impact of MASLD and MASLD‐related comorbidities on post‐LT outcomes is critical as rates of obesity continue to increase and as MASLD represents the fastest growing indication for LT in the United States [17, 22]. The findings in this study align with the current consensus on LT for MASLD etiology, which suggests that patients undergoing LT for MASLD have higher comorbid risk profiles [12, 23]. Despite this, our study found that MASLD LT recipients had reduced 90‐day readmission and morbidity rates. One explanation for this may be the reduced rates of cardiovascular complications in MASLD LT recipients. It is well established that cardiovascular disease is a major source of morbidity and mortality after LT, especially in those with alcohol associated liver disease who may develop alcohol associated cardiomyopathy and are more likely to develop stress‐induced cardiomyopathy [24]. Although MASLD LT recipients may have increased metabolic syndrome comorbidities, it appears this does not translate into an increase in cardiovascular complications following LT [25].

Few studies have evaluated independent risk factors for adverse outcomes among MASLD LT recipients. Our study identified several risk factors conferring an increased risk of 90‐day morbidity and readmission in MASLD LT recipients, including CKD, need for dialysis, COPD, and a history of prior bariatric surgery. The impact of prior bariatric surgery on LT is controversial. One single‐center study performed a comparative study of patients with cirrhosis with a prior history of bariatric surgery and found that delisting/death on the waitlist, rate of transplantation, and intention‐to‐treat survival from listing to one year after LT were all worse in those with a prior history of bariatric surgery compared to those without bariatric surgery [25]. The authors suggested one explanation for the inferior outcomes may have been higher rates of malnutrition and sarcopenia in the bariatric surgery cohort. Conversely, a single‐center study found no difference in post‐LT outcomes among those with a prior history of bariatric surgery undergoing LT compared to those without bariatric surgery [26]. Both of these studies are plagued by small sample sizes inherent to a single‐center study as well as the heterogenous indications for LT. Our analysis of a national database among MASLD LT recipients suggests those with a prior history of bariatric surgery are at a higher risk for postoperative readmission and morbidity. We hypothesize that this may be a result of several factors, including increased technical complexity secondary to prior abdominal surgery, subtherapeutic dosing of immunosuppression due to inadequate malabsorption, and increased malnutrition/sarcopenia in bariatric surgery recipients, as suggested by Idriss et al. LT recipients with MASLD and with a prior history of bariatric surgery should be closely monitored in the post‐LT setting.

The obesity paradox is an established phenomenon in which patients with obesity have improved outcomes compared to non‐obese patients. The obesity paradox has been previously demonstrated in those with advanced liver disease as well as LT recipients, although this was limited to long‐term graft and patient survival and did not incorporate short‐term morbidity and readmissions [8, 27]. In our analysis, although MASLD LT recipients with class 3 obesity had more significant comorbidities compared to MASLD LT recipients without class 3 obesity, those with BMI ≥ 40 had a decreased risk of 90‐day morbidity following LT and similar rates of readmission. Further, MASLD LT recipients had lower rates of 90‐day morbidity and readmission compared to those undergoing LT for alcohol associated liver disease, despite having a higher percentage of recipients with class 3 obesity (11.5% vs. 3.8%). One explanation for this “obesity paradox” is that obesity provides some degree of nutritional reserve that allows LT recipients to withstand the insult associated with a LT. Our findings go a step further and demonstrate that among those with class 3 obesity, MASLD LT recipients have improved short term outcomes relative to non‐MASLD LT recipients, suggesting that a diagnosis of MASLD itself is advantageous in the post operative LT period.

Along similar lines, there is strong evidence that increased body fat confers increased rates of liver cancer, which implies a relationship between MASLD and the development of liver cancer [28]. Our analysis suggests that MASLD LT recipients with liver cancer have improved 90‐day outcomes compared to MASLD LT recipients without liver cancer. This is not surprising, as this is likely explained by lower native MELD scores (vs. MELD exception points) in those with liver cancer, indicating a reduced burden of liver disease. Future analysis should investigate whether these results persist after adjusting for MELD score. However, our analysis among LT recipients with a diagnosis of liver cancer again demonstrated that despite a higher comorbid risk profile among MASLD LT recipients with liver cancer relative to non‐MASLD LT recipients with liver cancer, those with MASLD had either improved or similar 90‐day outcomes. These data reiterate the protective effect of a MASLD diagnosis on post‐LT morbidity.

Despite expectations that higher BMI and diabetes rates would predispose MASLD recipients to increased wound infections following LT, our data show that 90‐day infection rates—aggregating superficial, deep space, and surgical site infections—were actually lower in the MASLD group compared to non‐MASLD recipients. This observation is emblematic of the obesity paradox previously described in transplant outcomes, where increased adiposity may confer some protective effects against certain postoperative complications, despite higher baseline metabolic risk. One plausible explanation for lower wound infection rates among MASLD patients is the notably higher prevalence of smoking in non‐MASLD recipients, since smoking is an established risk factor for poor wound healing and infectious complications post‐surgery. These findings support the notion that patient selection, metabolic disease burden, and behavioral risk factors collectively influence postoperative infection risks in this population.

The limitations of this study should be considered, including its retrospective and observational nature, which may contribute to unintended selection bias. Second, the ICD‐10 coding system is a potential limitation as it is subject to coding attribution error, although it is the accepted gold standard for global medical coding. Third, the lack of LT‐specific information, including laboratory values (i.e., MELD score) and donor health factors, is a further limitation of the NRD. Despite these limitations, this study represents one of the largest cohort series studies containing recipient readmission information after LT.

## 5 Conclusion

Although LT recipients with MASLD represent a higher‐risk surgical population by comorbidity status, short‐term outcomes, including 90‐day readmission and morbidity, are superior to LT recipients with non‐MASLD liver disease. This may be related to a reduced risk of cardiovascular complications post‐LT as well as a “protective” effect conferred through increased rates of obesity (i.e., obesity paradox). Finally, relative to non‐MASLD LT recipients, MASLD etiology appears to normalize 90‐day outcomes among patients with obesity and liver cancer despite an increased comorbid risk profile.

## Author Contributions

Zachary Leslie participated in research design, performance of research, data analysis, and writing of the paper. Thomas Leventhal, Sean Nguyen, David Leishman, Cheyenna Espinoza, Eric Wise, Sayeed Ikramuddin, and Raja Kandaswamy participated in data analysis and writing of the paper. Abraham J. Matar participated in research design, performance of research, data analysis, and writing of the paper.

## Disclosure

The authors have nothing to disclose.

## Conflicts of Interest

The authors declare no conflicts of interest.

## Supporting information




**Table S1**. List of International Classification of Diseases and Related Comorbidities, 10th revision, used in this study. ICD – International Classification of Diseases.
**Table S2**. Characteristics and outcomes among MASLD LT recipients stratified by liver cancer status within the NRD between 2016 and 2022.
**Table S3**. Characteristics and outcomes among LT recipients with a diagnosis of liver cancer stratified by MASLD versus non‐MASLD within the NRD between 2016 and 2022.
**Figure S1**. CONSORT diagram for patients included in this study of the Nationwide Readmissions Database from 2016 to 2022.
Figure S2: Distributions of body mass index (BMI, kg/m^2^) across the Nationwide Readmissions Database from 2016 to 2022 for patients with and without metabolic‐associated steatotic liver disease who underwent liver transplantation.

## Data Availability

The data that support the findings of this study are available from the corresponding author upon reasonable request.
