# Supplementary material for: Ninety‐Day Readmission and Morbidity Following Liver Transplantation for MASLD
Source: Clin Transplant. 2026 Feb 4;40(2):e70461. doi: 10.1111/ctr.70461 (PMC12873555; doi:10.1111/ctr.70461)

**SUPPLEMENTARY**

**Supplementary Table 1.** List of international classification of diseases and related comorbidities, 10th revision, used in this study. ICD – International classification of diseases.

| **Variable** | **ICD-10 Code** |
| --- | --- |
| Liver cancer | C22 |
| Alcoholic liver cirrhosis | K7031, K7040, K7030, K7011, K7010 |
| Chronic hepatitis C | B182, B192, B171 |
| Metabolic dysfunction-associated steatotic liver disease | K7581 |
| Metabolic associated alcoholic liver disease | E6601 and K70 |
| Fibromyalgia | M797 |
| Rheumatoid arthritis | M069 |
| Chronic heart failure | I50, I130, I131, I132, I110 |
| Coronary heart disease | I251 |
| Long term NSAID | Z791, Z7982 |
| Anticoagulant therapy | Z7901, Z902 |
| COPD | J449 |
| Presence coronary graft | Z951, Z955 |
| CKD | N18, I29 |
| Hypertension | I10 |
| Hyperlipidemia | E78 |
| Morbid obesity | E6601 |
| Smoker | F1721 |
| GERD | K21 |
| Type 2 diabetes | E10, E11 |
| Prior bariatric surgery | Z9884 |
| B12 anemia | D50, D51, D52, D649 |
| Tobacco nicotine dependence | Z720, F172 |
| Opiod use disorder | F119, F112, F1121, F1120 |
| Cannabis use | F1120, F12988 |
| Anxiety disorder | F410, F411, F418, F419 |
| Dialysis | Z992 |
| 90-day acute renal failure | N990 |
| 90-day sepsis | R65, A41, B377, P36 |
| 90-day blood transfusion | 302 |
| 90-day UTI | N390 |
| 90-day PE | I82 |
| 90-day DVT | I26 |
| 90-day cardiac arrest | I46 |
| 90-day MI | I21 |
| 90-day infection | A419, T8613, N390, T814XX, T868, T80211  A084, T857, B349, J069, T827, T835, T8743,  T80212, T8469, T835, N995, B348, T8579, T802 |
| 90-day ventilator dependency | 5A1935Z, 5A1945Z, 5A1955Z |
| 90-day pneumonia | J15, J18 |

**Supplementary Table 2.** Characteristics and outcomes among MASLD LT recipients stratified by liver cancer status within the NRD between 2016 - 2022.

| **Variable** | **Non-liver cancer (n=8858)** | **Liver cancer (n=2376)** | **p-value** |
| --- | --- | --- | --- |
| **Demographics** | | | |
| Age | 59.17 (+-8.66) | 63.44 (+-6.5) | <0.05 |
| Year | 3.32 (+-1.92) | 3.36 (+-1.92) | 0.6 |
| Resident | 6770 (76.42%) | 1772 (74.57%) | 0.07 |
| Female sex | 4136 (46.69%) | 762 (32.07%) | <0.05 |
| Medicare | 3341 (37.71%) | 1097 (46.17%) | <0.05 |
| Medicaid | 763 (8.61%) | 117 (4.92%) | <0.05 |
| Private insurance | 4269 (48.19%) | 1041 (43.81%) | <0.05 |
| **Hospital/operative factors** | | | |
| Small bed size | 357 (4.03%) | 36 (1.52%) | <0.05 |
| Medium bed size | 922 (10.41%) | 192 (8.08%) | <0.05 |
| Large bed size | 6205 (70.04%) | 1749 (73.61%) | <0.05 |
| High volume (>90) | 3383 (38.19%) | 1063 (44.74%) | <0.05 |
| Elective | 1321 (14.91%) | 446 (18.77%) | <0.05 |
| Median patient zip code income level (1-4) | 2195 (24.78%) | 491 (20.66%) | <0.05 |
| City size of patient residence (1-7) | 1622 (18.31%) | 591 (24.87%) | <0.05 |
| Comorbidities |  |  |  |
| Charlson comorbidity index | 2.8 (+-1.81) | 4.5 (+-1.65) | <0.05 |
| Chronic heart failure | 714 (8.06%) | 136 (5.72%) | <0.05 |
| Coronary artery disease | 1181 (13.33%) | 480 (20.2%) | <0.05 |
| Long term NSAID | 680 (7.68%) | 316 (13.3%) | <0.05 |
| Anticoagulant therapy | 476 (5.37%) | 148 (6.23%) | 0.11 |
| COPD | 387 (4.37%) | 74 (3.11%) | <0.05 |
| Presence coronary graft | 427 (4.82%) | 194 (8.16%) | <0.05 |
| CKD | 2646 (29.87%) | 316 (13.3%) | <0.05 |
| Hypertension | 2969 (33.51%) | 1247 (52.48%) | <0.05 |
| Hyperlipidemia | 2647 (29.88%) | 942 (39.64%) | <0.05 |
| Morbid obesity | 1082 (12.21%) | 236 (9.93%) | <0.05 |
| Smoker | 278 (3.14%) | 64 (2.69%) | 0.3 |
| GERD | 2354 (26.57%) | 663 (27.9%) | 0.19 |
| Type 2 diabetes | 5023 (56.7%) | 1629 (68.56%) | <0.05 |
| Prior bariatric surgery | 249 (2.81%) | 19 (0.8%) | <0.05 |
| Tobacco nicotine dependence | 425 (4.8%) | 99 (4.17%) | 0.22 |
| Dialysis | 363 (4.1%) | 15 (0.63%) | <0.05 |
| **Outcomes** | | | |
| 90-day readmission | 5559 (62.75%) | 993 (41.79%) | <0.05 |
| Mortality | 119 (1.34%) | 32 (1.35%) | 0.93 |
| 90-day acute renal failure | 214 (2.42%) | 58 (2.44%) | 0.9 |
| 90-day sepsis | 2122 (23.95%) | 303 (12.75%) | <0.05 |
| 90-day blood transfusion | 3314 (37.41%) | 693 (29.16%) | <0.05 |
| 90-day UTI | 1955 (22.07%) | 236 (9.93%) | <0.05 |
| 90-day PE | 760 (8.58%) | 164 (6.9%) | <0.05 |
| 90-day DVT | 114 (1.29%) | 24 (1.01%) | 0.28 |
| 90-day cardiac arrest | 135 (1.52%) | 27 (1.14%) | 0.2 |
| 90-day MI | 293 (3.31%) | 57 (2.4%) | <0.05 |
| 90-day infection | 3048 (34.41%) | 385 (16.2%) | <0.05 |
| 90-day ventilator dependency | 1786 (20.16%) | 347 (14.6%) | <0.05 |
| 90-day pneumonia | 1193 (13.47%) | 186 (7.83%) | <0.05 |
| 90-day morbidity | 6128 (69.17%) | 1245 (52.4%) | <0.05 |
| LOS | 13.37 (+-18.39) | 10.91 (+-14.27) | <0.05 |
| Total charges | 318479.39 (+-447815.89) | 368881.65 (+-380598.78) | <0.05 |
| 90-day cardiovascular event | 1176 (13.27%) | 247 (10.39%) | <0.05 |

**Supplementary Table 3.** Characteristics and outcomes among LT recipients with a diagnosis of liver cancer stratified by MASLD vs. non-MASLD within the NRD between 2016 - 2022.

| **Variable** | **Non-MALSD (n=9715)** | **MASLD (n=2376)** | **p-value** |
| --- | --- | --- | --- |
| **Demographics** | | | |
| Age | 59.06 (+-13.3) | 63.44 (+-6.5) | <0.05 |
| Year | 2.72 (+-1.97) | 3.36 (+-1.92) | <0.05 |
| Resident | 7687 (79.12%) | 1772 (74.57%) | <0.05 |
| Female sex | 2150 (22.13%) | 762 (32.07%) | <0.05 |
| Medicare | 3864 (39.77%) | 1097 (46.17%) | <0.05 |
| Medicaid | 1307 (13.45%) | 117 (4.92%) | <0.05 |
| Private insurance | 3943 (40.58%) | 1041 (43.81%) | <0.05 |
| **Hospital/operative factors** | | | |
| Small bed size | 265 (2.73%) | 36 (1.52%) | <0.05 |
| Medium bed size | 841 (8.66%) | 192 (8.08%) | 0.38 |
| Large bed size | 7533 (77.54%) | 1749 (73.61%) | <0.05 |
| High volume (>90) | 4019 (41.37%) | 1063 (44.74%) | <0.05 |
| Elective | 1910 (19.66%) | 446 (18.77%) | 0.32 |
| Median patient zipcode income level (1-4) | 2249 (23.15%) | 491 (20.66%) | 0.32 |
| City size of patient residence (1-7) | 2949 (30.35%) | 591 (24.87%) | <0.05 |
|  |  |  |  |
| Charlson comorbidity index | 3.97 (+-1.57) | 4.5 (+-1.65) | <0.05 |
| Fibromyalgia | 40 (0.41%) | 30 (1.26%) | <0.05 |
| Rheumatoid arthritis | 68 (0.7%) | 30 (1.26%) | <0.05 |
| Chronic heart failure | 352 (3.62%) | 136 (5.72%) | <0.05 |
| Coronary artery disease | 1214 (12.5%) | 480 (20.2%) | <0.05 |
| Long term NSAID | 909 (9.36%) | 316 (13.3%) | <0.05 |
| Anticoagulant therapy | 466 (4.8%) | 148 (6.23%) | <0.05 |
| COPD | 618 (6.36%) | 74 (3.11%) | <0.05 |
| Presence coronary graft | 367 (3.78%) | 194 (8.16%) | <0.05 |
| CKD | 1065 (10.96%) | 316 (13.3%) | <0.05 |
| Hypertension | 4554 (46.87%) | 1247 (52.48%) | <0.05 |
| Hyperlipidemia | 1621 (16.68%) | 942 (39.64%) | <0.05 |
| Smoker | 594 (6.11%) | 64 (2.69%) | <0.05 |
| GERD | 2048 (21.08%) | 663 (27.9%) | <0.05 |
| Type 2 Diabetes | 3072 (31.62%) | 1629 (68.56%) | <0.05 |
| Prior bariatric surgery | 27 (0.28%) | 19 (0.8%) | <0.05 |
| B12 anemia | 1348 (13.87%) | 291 (12.25%) | <0.05 |
| Tobacco nicotine dependence | 814 (8.38%) | 99 (4.17%) | <0.05 |
| Opioid use disorder | 145 (1.49%) | 2 (0.08%) | <0.05 |
| Cannabis use | 103 (1.06%) | 1 (0.04%) | <0.05 |
| Anxiety disorder | 928 (9.55%) | 183 (7.7%) | <0.05 |
| Dialysis | 127 (1.31%) | 15 (0.63%) | <0.05 |
| **Outcomes** | | | |
| 90-day readmission | 4169 (42.91%) | 993 (41.79%) | 0.34 |
| Mortality | 191 (1.97%) | 32 (1.35%) | 0.05 |
| 90-day acute renal failure | 245 (2.52%) | 58 (2.44%) | 0.88 |
| 90-day sepsis | 1483 (15.26%) | 303 (12.75%) | <0.05 |
| 90-day blood transfusion | 3127 (32.19%) | 693 (29.16%) | <0.05 |
| 90-day UTI | 743 (7.65%) | 236 (9.93%) | <0.05 |
| 90-day PE | 637 (6.56%) | 164 (6.9%) | 0.51 |
| 90-day DVT | 170 (1.75%) | 24 (1.01%) | <0.05 |
| 90-day cardiac arrest | 135 (1.39%) | 27 (1.14%) | 0.39 |
| 90-day MI | 212 (2.18%) | 57 (2.4%) | 0.47 |
| 90-day infection | 1739 (17.9%) | 385 (16.2%) | 0.05 |
| 90-day ventilator dependency | 1331 (13.7%) | 347 (14.6%) | 0.26 |
| 90-day pneumonia | 818 (8.42%) | 186 (7.83%) | 0.36 |
| 90-day morbidity | 5304 (54.59%) | 1245 (52.4%) | 0.05 |
| 90-day cardiovascular event | 1034 (10.64%) | 247 (10.39%) | 0.73 |
| LOS | 12.39 (+-18.6) | 10.91 (+-14.27) | <0.05 |
| Total charges | 419564.65 (+-552608.42) | 368881.65 (+-380598.78) | <0.05 |

**Supplementary Figure 1.** CONSORT diagram for patients included in this study of the Nationwide Readmissions Database from 2016 to 2022.


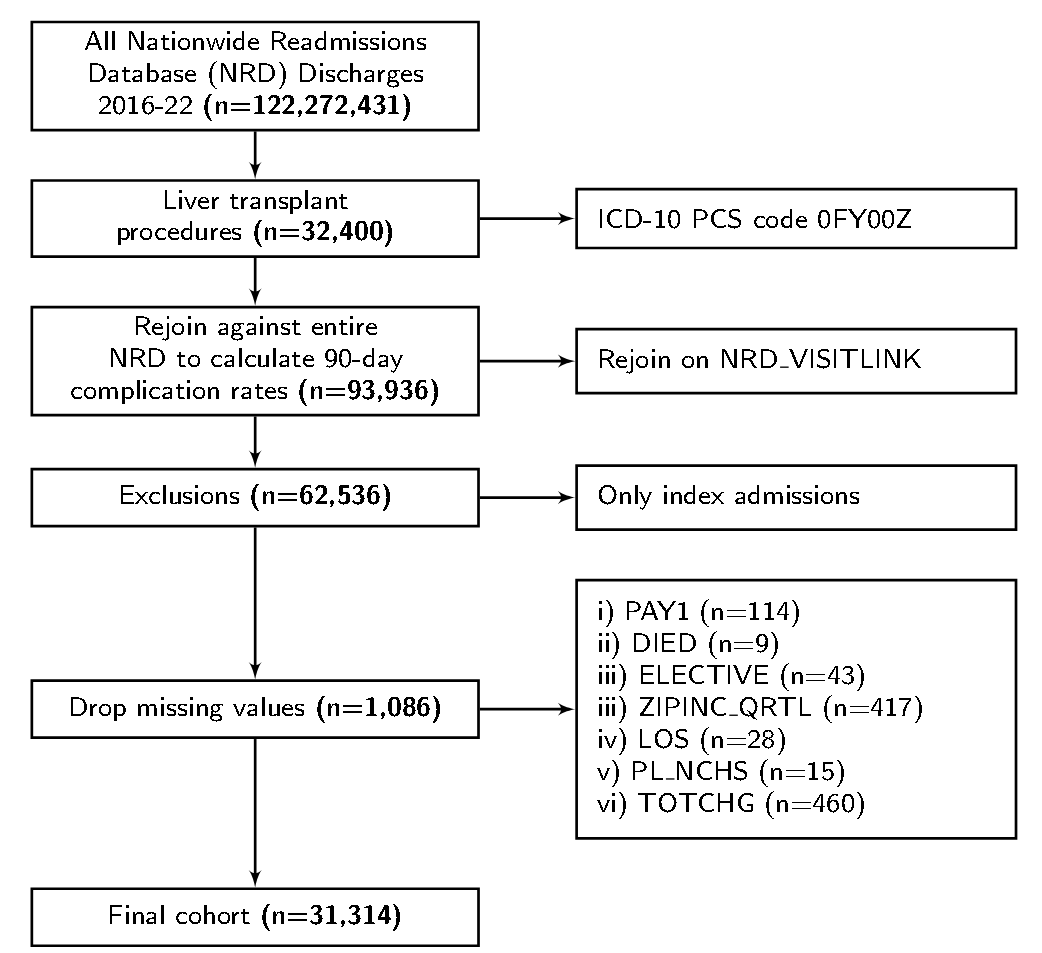


ICD-10 = International classification of diseases, 10th revision. PAY1 = data field for insurance payer status in the Healthcare cost and Utilization Project (HCUP) database, DIED = data field for mortality status in the HCUP database, data field for elective status in the HCUP database, ZIPINC_QRTL = data field for median zip code income level in the HCUP database, LOS = length of stay data field in the HCUP database, PL_NCHS = city size of patient residence data field in the HCUP database, TOTCHG = total charges data field in the HCUP database.

Supplemental figure 2: Distributions of Body Mass Index (BMI, kg/m^2^) across the Nationwide Readmissions Database from 2016 to 2022 for patients with and without Metabolic-associated steatotic liver disease who underwent liver transplantation.


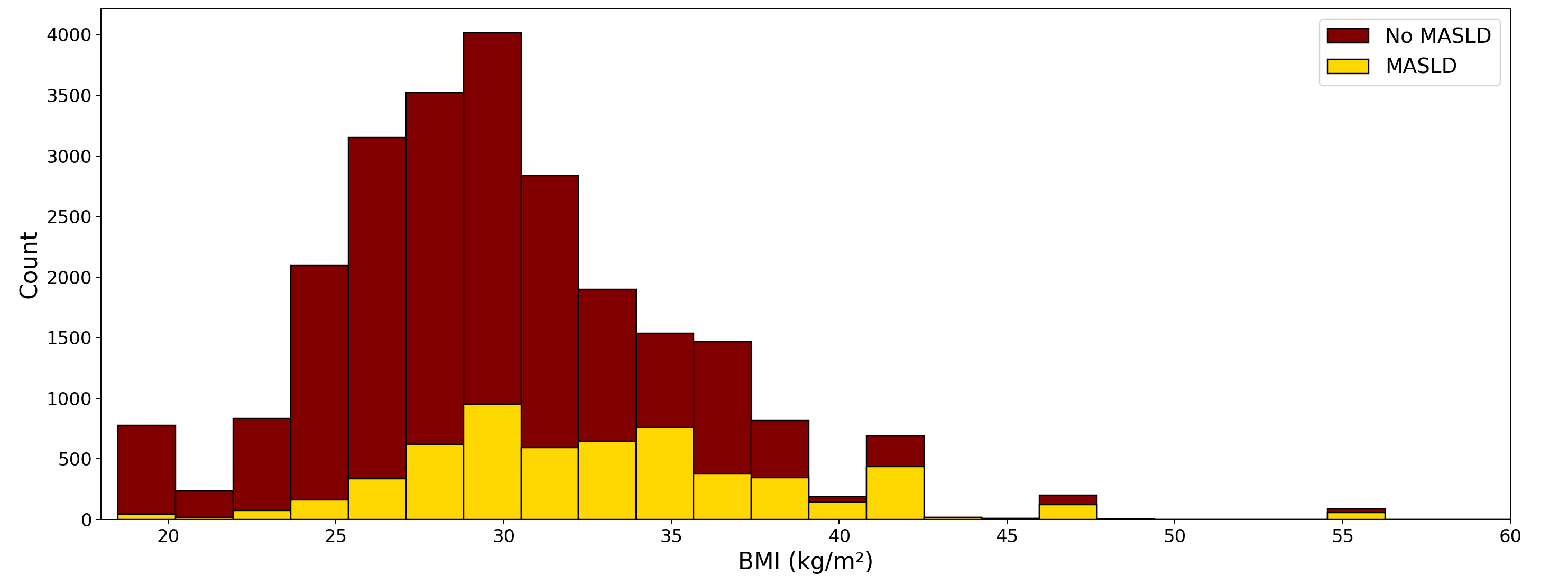

Supplement: Supplementary file 1 — Table S1. List of International Classification of Diseases and Related Comorbidities, 10th revision, used in this study. ICD – International Classification of Diseases. Table S2. Characteristics and outcomes among MASLD LT recipients stratified by liver cancer status within the NRD between 2016 and 2022. Table S3. Characteristics and outcomes among LT recipients with a diagnosis of liver cancer stratified by MASLD versus non‐MASLD within the NRD between 2016 and 2022. Figure S1. CONSORT diagram for patients included in this study of the Nationwide Readmissions Database from 2016 to 2022. Figure S2: Distributions of body mass index (BMI, kg/m2) across the Nationwide Readmissions Database from 2016 to 2022 for patients with and without metabolic‐associated steatotic liver disease who underwent liver transplantation. [file CTR-40-e70461-s001.docx]
